# The Ethical Side of Professional Secrecy in Relation to Venereal Disease

**Published:** 1907-10

**Authors:** 


### The Ethical Side of Professional Secrecy in Relation to Venereal Disease.

Venereal disease is without doubt considered by modern medical science as one of the greatest scourges the human race has to combat. The one formerly considered comparatively trivial, gonorrhea, is, at least in its results, the more to be dreaded of the two. The sin of the infected is constantly visited upon the innocent, and entails on countless thousands sterility, insanity, and a host of maladies, to the very great danger to the state. Death would be more welcome than life to many sensitive and honorable persons polluted by these shameful diseases, accompanied by the knowledge that the evil may be transmitted to children. Thou shalt not murder, is the highest law, human or divine. Law, however, permits us to kill under certain circumstances. For instance, in self-defense. Abortion, criminal if committed with evil intent, is lawful if performed to save the mother's life. Now, if this great leading law, "Thou shalt not kill," is subject to limitation, it seems that the duty of physicians to conceal what they have learned of a patient's condition would be overshadowed by the (to them) undoubtedly paramount duty, viz., the saving of life and the preservation of health. It seems, also, from a lawyer's view of the case, that where the preserving of a patient's secrets conflicts with the higher duty to the individual or the state, the former should yield. In law, he who aids or abets a crime is himself a criminal. Morally, he who consents to a wrong which he might prevent, is a criminal. He who communicates a venereal disease inflicts a very great bodily injury upon that person and a wrong to the state. The law justifies homicide committed in resisting great bodily injury, and would thus justify the killing in order to prevent wilful infection.

The articles of war of the United States army expressly provide that an officer or soldier committing an outrage on a woman in an invaded district may, if caught in the act, be shot on the spot, or may be punished by courtmartial by death. Poisoning weapons or wells or sending out infection in any way, is forbidden in civilised warfare. If this is true with reference to an enemy, why is not poisoning the springs of life in those most entitled to love and protection equally so? Upon what ground is any one who intends to deliberately spread contagion entitled to any protection? This is, indeed, a hard question, and much more difficult from the side of the physician than that of a lawyer. It would seem that the physician, knowing that an infected patient is about to carry his contagion to a pure person, and perhaps to persons unborn, is justified in both law and morals in preventing the proposed wrong by disclosing his knowledge, if no other way is open.

Under our system of jurisprudence, medical men reporting venereal diseases might be held liable in two ways: First, it is slanderous to say, and libelous to write, that a person is so affected; for both slander and libel a civil action lies against the defamer, in which, as a general rule, the truth is a complete justification of defense. For libel, *i.e.*, written defamation, a criminal action also lies, to which it is a defense to show that the libelous matter is true and was published from good motives to justifiable ends.

